# Membrane Lesions and Reduced Life Span of Red Blood Cells in Preeclampsia as Evidenced by Atomic Force Microscopy

**DOI:** 10.3390/ijms24087100

**Published:** 2023-04-12

**Authors:** Ina Giosheva, Velichka Strijkova, Regina Komsa-Penkova, Sashka Krumova, Ariana Langari, Avgustina Danailova, Stefka G. Taneva, Tanya Stoyanova, Lora Topalova, Emil Gartchev, Galya Georgieva, Svetla Todinova

**Affiliations:** 1 Institute of Biophysics and Biomedical Engineering, Bulgarian Academy of Sciences, 1113 Sofia, Bulgaria; ina_gi@abv.bg (I.G.); vily_strij@abv.bg (V.S.); sashka.b.krumova@gmail.com (S.K.); arianalangari@abv.bg (A.L.); avgustina_danailova@abv.bg (A.D.); sgtaneva@gmail.com (S.G.T.); tanya.zh.stoyanova@gmail.com (T.S.); topalovaloram@gmail.com (L.T.); 2 University Obstetrics and Gynecology Hospital “Maichin Dom”, 1431 Sofia, Bulgaria; egartt@gmail.com; 3 Institute of Optical Materials and Technologies “Acad. Yordan Malinovski”, Bulgarian Academy of Sciences, 1113 Sofia, Bulgaria; 4 Department of Biochemistry, Medical University-Pleven, 5800 Pleven, Bulgaria or rkomsa@gmail.com (R.K.-P.); galia.georgieva-aleksandrova@mu-pleven.bg (G.G.)

**Keywords:** preeclampsia, red blood cells, atomic force microscopy, membrane roughness, membrane impairment, cell senescence

## Abstract

Preeclampsia (PE) presents with maternal de novo hypertension and significant proteinuria and is one of the leading causes of maternal and perinatal morbidity and mortality with unknown etiology. The disease is associated with inflammatory vascular response and severe red blood cell (RBC) morphology changes. This study examined the nanoscopic morphological changes of RBCs from PE women versus normotensive healthy pregnant controls (PCs) and non-pregnant controls (NPCs) applying atomic force microscopy (AFM) imaging. The results revealed that the membrane of fresh PE RBCs differed significantly from healthy ones by the presence of invaginations and protrusions and an increased roughness value (R_rms_) (4.7 ± 0.8 nm for PE vs. 3.8 ± 0.5 nm and 2.9 ± 0.4 nm for PCs and NPCs, respectively). PE-cells aging resulted in more pronounced protrusions and concavities, with exponentially increasing R_rms_ values, in contrast to the controls, where the R_rms_ parameter decreased linearly with time. The R_rms_, evaluated on a 2 × 2 µm^2^ scanned area, for senescent PE cells (13 ± 2.0 nm) was significantly higher (*p* < 0.01) than that of PCs (1.5 ± 0.2 nm) and NPCs (1.9 ± 0.2 nm). Furthermore, the RBCs from PE patients appeared fragile, and often only ghosts were observed instead of intact cells at 20–30 days of aging. Oxidative-stress simulation on healthy cells led to RBC membrane features similar to those observed for PE cells. The results demonstrate that the most pronounced effects on RBCs in PE patients are related to impaired membrane homogeneity and strongly altered roughness values, as well as to vesiculation and ghost formation in the course of cell aging.

## 1. Introduction

Preeclampsia (PE) is a pathological state associated with abnormal placentation and excessive maternal inflammatory vascular response [1]. It is one of the leading causes of maternal morbidity, preterm delivery, and fetal compromise. Preeclampsia can cause end organ damage, such as renal or liver impairment, hematological dysfunction, neurological excitability, and fetal growth restriction [2]. The disease is characterized by dysfunction of vascular endothelium caused by shallow infiltration of cytotrophoblast cells, narrowing arterioles leading to reduced blood flow, and ischemia [3]. PE is also associated with significant changes in the red blood cell (RBC) morphology and an increased fragility of the cell membrane [4,5].

In a healthy state, RBCs are biconcave-shaped cells with viscoelastic membranes and a high surface-to-volume ratio, facilitating their reversible deformability for gas exchange and oxygen delivery to all tissues. RBCs circulate between the lungs, where the oxygen level is high, exposing the cells to oxidative stress controlled by accelerated production of reducing equivalents and the peripheral tissues, including the placenta, where the oxygen pressure is low, and RBCs distort as they flow from small arterioles to the postcapillary venules where they switch to ATP production. The process is regulated by Band 3/AE1, which engages the pentose phosphate pathway (PPP) to reduce oxidative stress at high oxygen and switches glucose to energy production at low oxygen, required for passage through small capillaries. The composition of the membrane bilayer and the spectrin network determine the biconcave morphology of healthy RBCs and their elastic and rheological features. The high RBC deformability eventually determines erythrocyte survival, and it is closely related to the cell’s cytoskeleton, transmembrane transporters such as band 3/AE1, aquaporin, and the interactions of the membrane protein components with the glycolytic and redox enzymes of RBCs [6]. The cytoskeleton and the membrane are connected by protein complexes that participate in horizontal and vertical interactions [7]. These junctional complexes largely contribute to maintaining the biconcave RBC shape, elasticity, and stability [8,9]. Reduced deformability related to changes in the cell shape, membrane stiffness modification, and/or cytosol composition may occur in a variety of pathological conditions, and are also part of RBC aging [10].

During their lifespan, RBCs are exposed to many stressors. It is well-known that pregnancy increases oxidative stress, a phenomenon related to a systemic inflammatory response that results in high amounts of circulating reactive oxygen species, especially at the end of the first trimester [11].

The oxidative aggression towards the endothelium, leading to inflammation and subsequent increase in vascular resistance, and the corresponding rise in blood pressure, is suggested as one of the possible mechanisms for PE [12,13]. Oxidative stress biomarkers have been detected in the blood of women with PE [14], and it was shown that the RBC antioxidative defense pathway is markedly reduced in conditions of low oxygen [15]. Significantly increased levels of abnormal RBCs, as schistocytes and echinocytes, were found in women with PE in contrast to pregnant women without obstetric complications [4]. A strong relationship between PE and increased RBC distribution width (RDW), packed cell volume (PCV), and mean corpuscular volume (MCV) was observed [16]. Moreover, RBCs with higher volume and hemoglobin content are more likely to undergo hemolysis in vivo [17].

In recent years, numerous studies on the structural and mechanical properties of healthy cells and RBCs from patients with different pathological states have been conducted using atomic force microscopy (AFM) [18,19,20]. AFM is a powerful method of high resolution to analyze the topography and mechanics of biological samples due to its ability to image surfaces at the nanometer scale. AFM operates on the principle of surface sensing through the physical interaction of a cantilever tip with the molecules on the cell surface [21]. RBC topology [22], membrane roughness [23], and elasticity [24] were recognized as important predictors for various disorders and pharmacological treatments [25]. Our recent study also revealed specific morphometric features of young and senescent RBCs derived from women with early pregnancy losses that distinguish them from those of non-pregnant and pregnant controls in the first trimester of pregnancy [26].

The current work aimed to determine the nanostructural changes of RBCs derived from women with PE relative to normotensive pregnant subjects in the third trimester of pregnancy by AFM imaging. The presented results reveal the strong influence of this disorder on the morphometric characteristics of RBCs that could be used as a prognostic approach in the future.

## 2. Results

### 2.1. Patient Characteristics

Table 1 summarizes the hematological parameters determined for RBCs obtained from the studied groups of women. Some of the hematological parameters were outside the reference limits for more than half of the studied PE patients; nevertheless, the average values of the main hematological indices were in the reference range (Table 1).

Of the presented cases, five had severe preeclampsia, requiring early delivery.

### 2.2. Morphology and Nanostructure of Freshly Isolated RBCs

RBC morphology is one of the factors related to the clinical state [27]. During aging, RBCs lose their metabolic capacity and change their shape from the biconcave discoid shape (discocyte) typical of young cells to spiculated (echinocytes) and, at a later stage, spherical shape. Typical images of each morphological type, recorded by optical microscopy and AFM, are presented in Figures S1 and S2.

The relative contribution of the three morphological types of fresh RBCs in all groups under study is presented in Table 2, where it can be seen that the discocytes largely prevailed, followed by speculated cells and spherocytes, detected only for patients with PE.

The main dimensions (i.e., diameter, D; height, H; and volume, V) for fresh RBC estimated from the recorded AFM images are presented in Table 3. The cross-sectional profiling of AFM images also allowed for precise characterization of the RBC concavity and determination of depth, h_2_ (Figure S3). Our analysis showed that the average RBC diameter was about 8.1 µm for NPC and PC cells (Table 3), while in PE its value was statistically higher (8.5 µm) compared to the two control groups. No statistical difference was found between the maximum height (H) in the PE and PC groups. However, it is worth noting that the mean height of NPC erythrocytes was lower than that of PCs and patients with PE. The volume of patients’ cells was significantly higher as compared to those of NPC and PC cells, mainly as a result of the enlarged diameter. Our results showed that the ratio of the maximum height and the depth of concavity, H/h_2_, determined for the biconcave shape [28] had values close to those for PC and NPC cells, indicating a maximally high surface-to-volume ratio and an H/h_2_ ratio for PE cells that was twice as high (Table 3).

**Figure 1:**
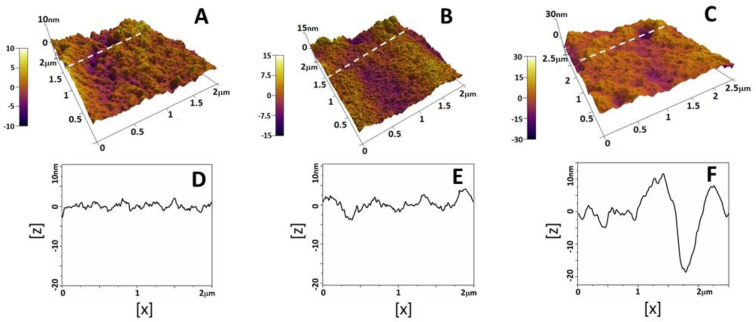
3D high-resolution images of membrane nanostructure of freshly isolated RBCs from non-pregnant controls (NPCs, (**A**)) and pregnant controls (PCs, (**B**)) and from patients with preeclampsia (PE, (**C**)). The height profiles defined by the dashed lines on panels (**A**–**C**) are presented on panels (**D**–**F**), respectively.

Next, we determined the roughness of the RBC membrane. The surface of healthy NPC and PC erythrocytes appeared homogeneous with a gently pleated membrane (Figure 1A,B). In contrast, the surface of fresh PE RBCs was heterogeneous and characterized by invaginations and protrusions located unevenly on the cell surface (Figure 1C), resulting in an enhanced roughness value (Table 2). In some cases, vesicles were also observed. The average number of cavities was about 0.5/µm^2^, and their sizes were 0.06 ± 0.02 µm in depth and 0.4 ± 0.02 µm in diameter.

### 2.3. Alteration of Morphology and Nanostructure along the Aging of RBCs

**Figure 2:**
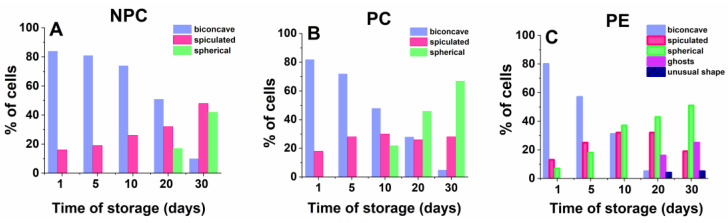
Histogram of the relative contribution of RBCs assigned to the main morphological classes, along with the senescence of cells derived from healthy non-pregnant controls (NPCs, (**A**)); healthy pregnant controls (PCs, (**B**)), and patients with preeclampsia (PE, (**C**)). Each morphological type is presented as a percentage of the total number of cells.

We followed the change of the RBCs’ shape and membrane nanostructure during cellular aging. The data analysis revealed that discocytes were the dominant cell type up to the 20th day of storage time for the NPC group. A lower percentage of spiculocytes was also observed, which gradually increased with age (Figure 2A). After the 20th day, the number of biconcave RBCs dramatically decreased at the expense of spiculocytes and spherocytes that become prevalent at the end of the follow-up period (Figure 2A). In the PC group, the morphological cell transformation occurred at a faster rate compared to NPCs. Spiculocytes had already increased by the 5th day of storage, and their relative abundance remained nearly constant for the monitoring period of 30 days. Spherocytes emerged after ten days of storage and gradually became a major morphological type as time progressed (Figure 2B).

**Figure 3:**
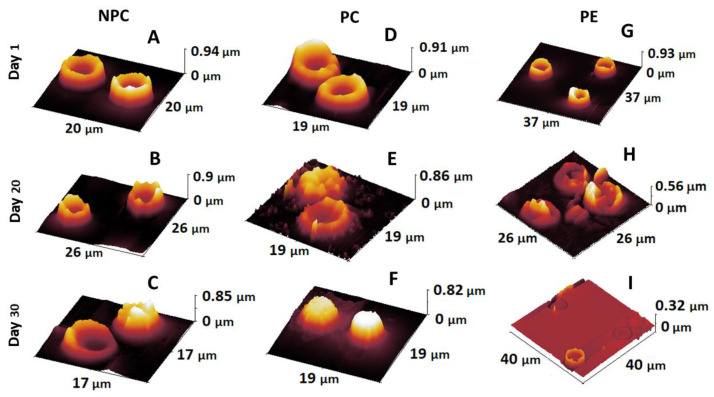
Selected 3D images of RBCs isolated from non-pregnant women (NPC, (**A**–**C**)), healthy pregnant women (PC, (**D**–**F**)), and patients with preeclampsia (PE, (**G**–**I**)). The images show morphological changes in aging cells over a storage period of 30 days.

**Figure 4:**
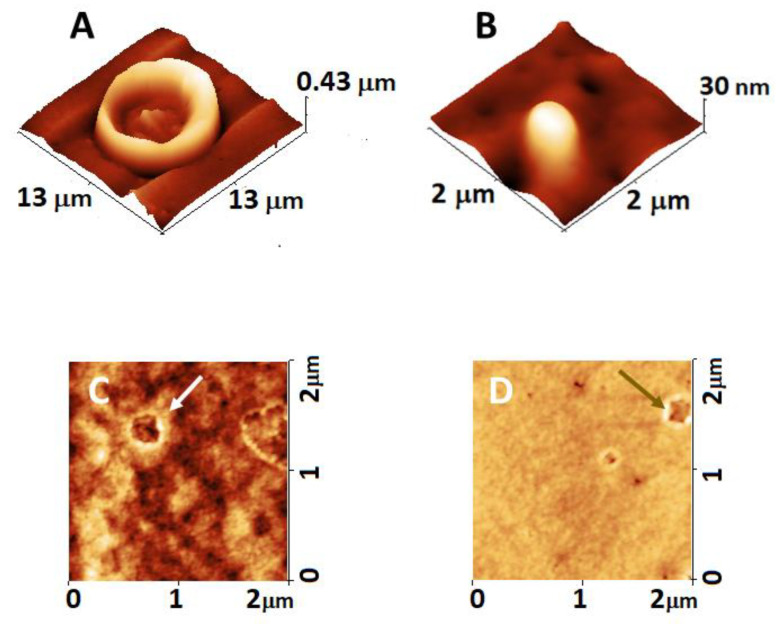
Example of 3D images of a whole RBC (**A**) and RBC membrane patch scanned in an area of 2 × 2 µm^2^ (**B**) from a patient with preeclampsia, representing the initial formation of vesicles. Panels (**C**,**D**) illustrate the disturbances on the membrane surface after vesicle extrusion (denoted with arrows). The scale bar in panel (**A**) is 13 µm, and in panels (**B**–**D**) is 2 µm.

The aging process of PE cells was faster than that of NPC and PC ones. The structural alteration of erythrocytes obtained from women with preeclampsia started at an earlier stage; the three morphological classes had an almost equal distribution by the 10th day (Figure 2C). Along the aging path, the proportion of RBCs with spiculated and spherical shapes increased, and on the 20th day, they were significantly more than the discocytes. The presence of cells with unusual forms (i.e., cells with irregular protrusion instead of normal concavity) was also observed in 16% of PE cases in this period; examples are shown in Figure 3H and Figure S4A. Of note, we discovered cells with abnormal form in three of the cases with severe PE. The initial formation of vesicles can also be seen in some images (Figure 4). In addition, the cells from most PE patients appeared very fragile, and often only ghosts (Figure S4B) were observed instead of intact cells at 20–30 days of storage (Figure 3).

The morphological alterations were analyzed in detail by AFM and selected 3D images of different periods of erythrocyte aging in the studied groups are presented in Figure 3. It can be seen that biconcave cells are present even after 30 days of storage of NPC cells (Figure 3A–C); biconcave cells, spiculocytes, and spherocytes were observed in aged PC cells (Figure 3B,C), while ghosts and cells with abnormal morphology were often observed on days 20 and 30 of the follow-up period in the PE group (Figure 3H,I).

**Figure 5:**
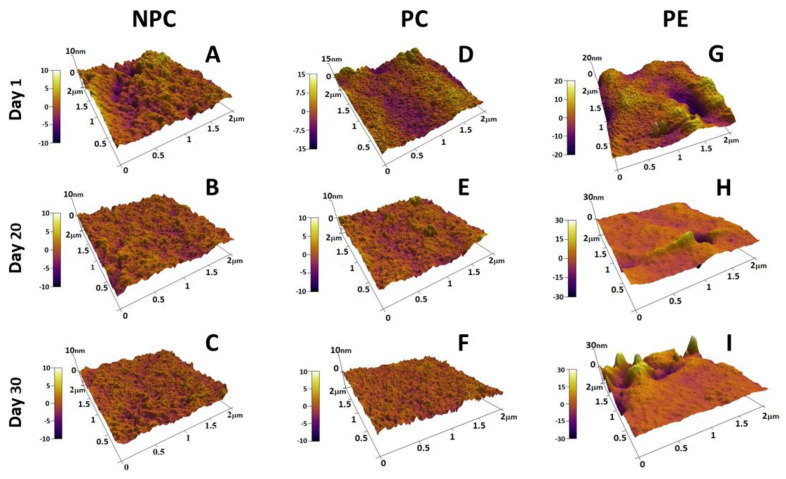
Selected 3D AFM images, representing the ultrastructural alteration of the membrane surface of RBCs isolated from patients with preeclampsia (PE (**G**–**I**)), non-pregnant controls (NPCs (**A**–**C**)), and pregnant controls (PCs (**D**–**F**)) during the cells’ senescence. The scanned area is 2 × 2 µm^2^.

The aging process during 30 days of storage affected the RBC membrane structure of the groups differently. The surface of healthy NPC and PC cells appeared homogeneous throughout the storage period with gradual smoothing out of the structure, particularly at the later stage (Figure 5A–F). In contrast, as noted above, the surface of PE cells was already characterized by the occurrence of protrusions and nanopores/invaginations in fresh RBC preparations (Figure 5G), which became even more drastic with cell senescence, resulting in more significant protrusions and more pronounced concavities (Figure 5H,I).

**Figure 6:**
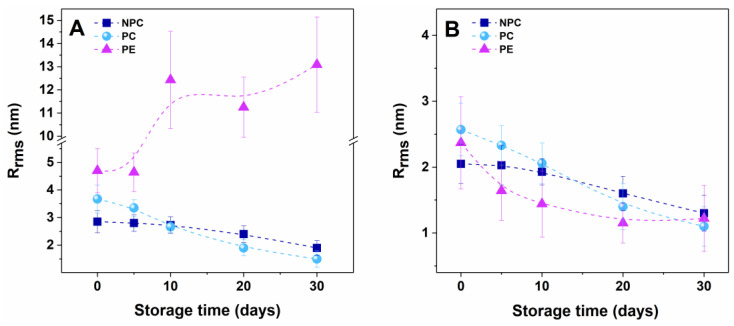
Membrane roughness value (R_rms_) determined as a function of storage time for RBCs derived from non-pregnant controls (NPCs), healthy pregnant controls (PCs), and patients with preeclampsia (PE). R_rms_ values were evaluated from topographic images with an area of either 2 × 2 µm^2^ (**A**) or 1 × 1 µm^2^ (**B**).

The membrane roughness as a function of the storage time for the two control groups decreased linearly. The R_rms_ values of young RBCs derived from PCs and NPCs were close for the entire 30-day monitoring period (Figure 6A) and gradually decreased. On the contrary, the R_rms_ value for PE cells was higher than the two control groups for freshly isolated RBC and further increased over the course of cells storage (Figure 6A). The roughness of senescent PE cells (13 ± 2.0 nm) was significantly higher (*p* < 0.01) than that of non-pregnant (1.9 ± 0.1 nm) and pregnant (1.5 ± 0.2 nm) controls. It should be pointed out that the R_rms_ values were evaluated from topographic images with an area of 2 × 2 µm^2^, and the evaluation was affected by the morphological disorders of the cell membrane. To avoid the influence of cavities and protrusions in this type of analysis, we determined the roughness of smaller areas (1 × 1 µm^2^) only in the sampling fields where no such membrane defects were detected (Figure 6B). The trend of R_rms_ dependence on cell senescence for the two control groups remained the same as the calculated roughness for the larger (2 × 2 µm^2^) scan field, but with lower values.

It should be noted that the measured roughness value depends on the scan area [29]. The average R_rms_ value for fresh PE cells was close to that of the PC group and higher than the NPC group, but no statistical difference was found concerning the latter. As the cells aged, the roughness of the PE group decreased exponentially, and as early as 10 days of the follow-up period, was significantly lower (*p* < 0.05) than the control ones, but after the 20th day, the roughness values were equal for the three groups.

### 2.4. Simulation of Oxidative Stress

**Figure 7:**
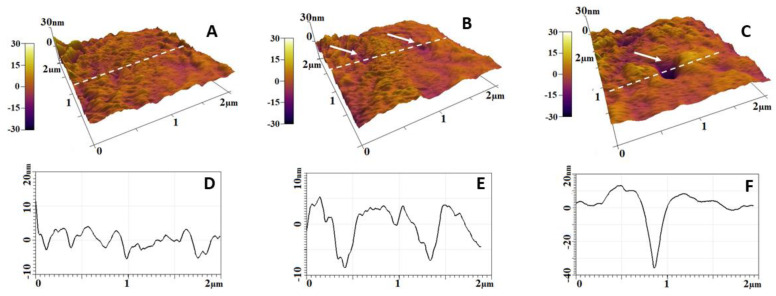
Representative 3D images of RBC membrane for non-treated, freshly isolated cells from healthy donors (**A**) and cells treated with 100 mM (**B**) and 200 mM (**C**) H_2_O_2_. The respective cross-sectional profiles defined by the dashed line at each panel are presented in panels (**D**–**F**). Arrows in panels B and C indicate the appearance of invaginations after treatment with H_2_O_2_.

One of the factors that may impair RBC membrane structure is oxidative stress. To test the vulnerability of RBCs to oxidative stress, we incubated newly isolated healthy RBCs with two concentrations (100 mM and 200 mM) of hydrogen peroxide (H_2_O_2_). Exposure to 100 mM H_2_O_2_ did not significantly affect the cell surface structure, and the appearance of small invaginations with a depth of 0.01 ± 0.003 µm was detected only for 15% of the treated cells (Figure 7B,E). Increasing the H_2_O_2_ concentration led to augmentation of the cavities’ size (Figure 7C,F). Their depth (0.04 ± 0.003 µm) was commensurable with that of the invaginations found in PE RBCs (0.06 µm) on the first day after their isolation (Figure 2C). However, it should be stressed that fewer than half of the cells treated with 200 mM H_2_O_2_ were affected, and in addition, the number of cavities was also lower (about one per 4 µm^2^) compared to fresh PE cells. The R_rms_ for 100 mM and 200 mM H_2_O_2_-treated cells were found to be 3.8 ± 0.4 nm and 4.2 ± 0.7 nm, respectively.

## 3. Discussion

Pregnancy as a particular stressor involves significant changes in many systems of the mother’s body, including blood and its components, which may appear to be pathological in a non-pregnant state. Deterioration in the microcirculatory environment of the bloodstream in PE can lead to significant alterations in the erythrocyte morphology [4].

To our knowledge, the present study provides the first nanostructural characterization of RBCs from preeclamptic patients compared to healthy non-pregnant and pregnant women, using the strength of AFM imaging.

Our initial goal was to detect the morphometric deviations in RBCs of PCs compared to NPCs and to differentiate them from the abnormalities of preeclamptic ones. The results show a significant difference between erythrocytes isolated from the three groups of women, both in the characteristics of young cells and in the progress of their aging.

### 3.1. Membrane Lesions and Vesiculation of PE Erythrocytes

One of the most prominent differences revealed for the PE cells compared to the controls was the occurrence of irregularly-shaped protrusions and invaginations resulting in impaired membrane homogeneity. This outcome suggested that defects in the skeleton trigger deformations of the RBC structure. The observed membrane defects might be associated with the rupture of local cytoskeleton–membrane bilayer junctions and, consequently, the inward sinking of the lipid bilayer at these sites. Any abnormalities or deficiencies of transmembrane proteins that link the bilayer to the membrane skeleton (such as Band 3, RhAG, ankyrin, or protein 4.2) and transduce the environment signal into metabolic changes lead to serious RBC deterioration or disorder [30]. Membrane integrity and flexibility are largely ensured by the interaction of Band 3 with the cytoskeleton through its N-terminal cytoplasmic attachment domain via mediator protein 4.2 and ankyrin. The plasma membrane plays an essential role in the RBCs’ functionality because of their incapability to replace destroyed membrane components by re-synthesis of new ones. Therefore, RBCs have acquired specific complex cellular mechanisms to precisely regulate their functions in the various environments in the bloodstream. One such mechanism of the membrane response could be the formation of vesicles, which in turn reduces flexibility and damages the overall erythrocyte integrity [31]. RBC deformability is an important hemorheological factor. The impaired RBCs’ deformability hinders their passage through smaller capillaries and impedes them from accomplishing their main goal of oxygen delivery. Thus, the cells’ reduced deformability can increase maternal blood viscosity [32] and impair oxygen transport to metabolically active tissues, such as the placenta. In line with this, Schauf et al. showed that RBCs from women with PE and/or intrauterine growth restriction have reduced deformability compared to those from women with uncomplicated pregnancies. This is more pronounced in cases with severe fetal or maternal complications [33]. The morphological changes detected as protrusions on the cell surface can be interpreted as an initial formation of vesicles. Such vesicles were seen on the images of PE samples. Indeed, the molecular dynamics model of the RBC membrane showed that weakened vertical connectivity between the lipid bilayer and the membrane skeleton is associated with the release of larger-sized vesicles upon lateral compression compared to normal RBCs [34].

### 3.2. Oxidative Stress as a Component of PE Development

Various factors such as deterioration of biochemical pathways by hypoxia, oxidative stress, immune response, and inflammatory process related to the extreme conditions of PE can lead to the observed abnormalities [11,35,36]. We hypothesized that oxidative stress is one of the possible causes of cell-membrane lesions and RBC impaired function.

In our previous work, the oxidative stress model demonstrated a significant reduction in biconcave cells and an increase in those with reduced functionality [26]. Herein, we focused on the structural changes of the plasma membrane that took place after the pretreatment of healthy erythrocytes with hydrogen peroxide. The progressive increase in membrane defects as a function of H_2_O_2_ concentration strongly suggests an impaired antioxidant enzyme machinery in RBCs from PE patients.

The function of red blood cells to transfer oxygen leads to significant damage from free radicals and oxidation. It involves oxidation of lipids and proteins including the main RBC proteins hemoglobin and Band 3 (which comprise 50% of membrane proteins). It was shown that oxidized hemoglobin produces non-functional aggregates—hemichromes. The binding of hemichromes to the *N*-terminus of Band 3 activates its clustering which in turn significantly reduces RBC oxygen-carrying capacity, anion transport regulation, and antioxidant capacity, ultimately leading to impaired red-blood-cell functionality [37]. Furthermore, biomarkers of oxidative stress have been shown in the blood of women with PE, as well as reduced red blood cell antioxidant capacity [14,15]. The work of Aris [38] supports this hypothesis—the authors showed that placental homogenates derived from patients with PE exhibit higher hydrogen peroxide production than those derived from healthy pregnant women. Furthermore, it was suggested that placental ischemia in PE decreases the antioxidant activity of glutathione peroxidase and catalase, essential enzymes of hydrogen peroxide degradation in RBCs [39].

### 3.3. Accelerated Aging of PE Erythrocytes

Another important result of our study was the finding of faster RBC aging for the PE group compared to the PC one, which is reflected in the degree of morphological changes and membrane roughness.

Morphologically, normal aging is associated with the gradual decrease of biconcave cells, at the expense of cells with reduced functionality. The senescence process is characterized by the reduced capacity of glycolysis and PPP pathways and, as a consequence, decreased production of ATP and reduced equivalents (antioxidative capacity), leading to morphological transformation. The ATP concentration inside the cells is one of the factors that play a crucial role in maintaining the connection between the spectrin network and the lipid bilayer [40], and its depletion triggers RBC morphological transformation from the normal discocyte to an echinocyte shape [41]. The aging of red blood cells is a complex process characterized by a decline in metabolic activity, cell shape transformation, significant changes in their membranes leading to an irreversible decrease in their flexibility and functionality, and the subsequent changes in RBCs’ rheological properties.

The accelerated aging of PC cells compared to NPCs was not surprising as it was already described by the faster kinetics of the RBC survival process during pregnancy [42].

In this study, even faster cell transformation for PE RBCs was established. Various factors influence cell modification, including ATP and antioxidative depletion, enzyme activity, and cholesterol enrichment. It should be noted that normal discocyte morphology could be lost in conditions when the structural properties of the cytoskeleton or the equilibrium between the two leaflets of the lipid bilayer are disturbed [43] as they are sensitive to external stimuli [44]. Oxidative and mechanical stress are strongly elevated in PE and are supposed to impair the properties of RBC, leading to lipid oxidation, Band 3 protein clustering, and changes in RBC adaptive capacity. Clustering of Band 3 protein leads to poor affinity to the cytoskeleton complex and consequently reduces membrane deformability and cell functionality. We can speculate that the faster aging, that we found, of red blood cells from PE patients, leading to their reduced functionality, may compromise the normal blood flow to the placenta and could be related to the pathogenesis of preeclampsia.

In addition to the earlier appearance of senescent cells, our results demonstrate an increased fragility of the plasma membrane resulting in ghost formation for the patients’ erythrocytes. It is reported that changes in the membrane or skeletal proteins result in reduced life span and premature removal of the RBCs from circulation [31]. The reduced RBC lifespan has been shown to play an important etiological role in patients with renal failure [45].

An interesting observation was the appearance of cells with unusual nanomorphology in some patients’ cases. One of the explanations could be the severity of preeclampsia, as these cases were related to more severe PE. However, it should be noted that only three of the five cases of severe PE had more pronounced changes in red blood cells. To further elucidate this phenomenon, more extensive research should be carried out. However, it can be assumed that a combined effect of oxidative stress, inflammatory components, mechanical aggression, and other disease-related factors may account for the abnormal membrane RBC lesions and cell deformation. Ostafiichuk et al. reported that the morphometric changes of RBCs characterized by an increase in their degenerative forms with limited functional capacity are related to the severity of the development of PE [46]. In line with our observation, Hernández et al. reported the presence of teardrop-shaped cells of variable size in the peripheral blood smear of patients with severe PE [47].

Cell aging is an additional factor that aggravates the biophysical properties of patients’ RBCs, including membrane integrity. RBC roughness is an important indicator of membrane integrity and cell deformability [48]. Many studies have revealed the dependence of the modifications of the RBC surface roughness from various disorders or aging conditions [18,22,28,49]. Our results showed a faster decrease in the roughness value (calculated at 1 × 1 µm^2^ scanned area) of cell membranes from patients, most dramatically expressed on the 10th day, during which period enhanced protrusions were detected in patient RBCs and which is most likely related to the vesiculation process presented in Figure 3H and Figure 4A–D. This process probably contributes to the exclusion of defective membrane parts. It has been suggested that the generation of extracellular vesicles represents a mechanism required to remove defective patches, i.e., non-functional proteins, thereby delaying the premature elimination of otherwise healthy RBCs [50,51].

The rate of membrane roughness reduction in the course of aging for PE cells was similar to that detected in RBCs derived from women with miscarriages [26] which indicates that similar factors could contribute to the rate of RBC roughness alteration in these different pregnancy complications.

In conclusion, the presented results demonstrate substantial morphological alterations of RBCs from preeclamptic women associated with structural rearrangements and progressive roughness alteration of the cell’s cytoskeleton.

One of the most pronounced effects on RBC morphology in PE patients compared to the control groups, was the presence of irregularly shaped protrusions and invaginations, resulting in impaired membrane homogeneity and strongly altered roughness values. Oxidation of healthy control RBCs produces a similar, but not identical effect, which is explained by the intact antioxidative capacity of the controls. The degree of oxidative and inflammatory damage generated in PE most probably contributes to the pathological changes observed in the RBCs derived from women affected by this pathology.

Based on our results, we can define the following biophysical markers for PE: (i) presence of concavities and protrusions on the membrane surface of freshly isolated cells; (ii) membrane microvesiculation; (iii) presence of RBCs with unusual morphology; and (iv) formation of ghosts at 20–30 days of the aging process.

A more detailed understanding of the pathological changes of RBCs in PE would help diagnose this pathology at an earlier stage. Our study provides a solid basis for future studies in this direction.

## 4. Materials and Methods

### 4.1. Study Groups and Ethic Statement

All subjects included provided written informed consent for the investigation. The study was approved by the Ethics Committee of the Medical University-Pleven (approval No. 404-KENID 22/10/15) and was performed in accordance with the Helsinki International ethical standards on human experimentation.

This study included 19 patients (mean age 27 ± 3 years) diagnosed with PE admitted to the Medical University-Pleven, and the Hospital of Obstetrics and Gynecology “Maichin Dom”, Medical University-Sofia, between February 2019 and November 2022. The diagnosis of PE was defined according to hypertension in pregnancy guidelines [52]. Twelve pregnant women (mean age 26 ± 3 years) in the third trimester of pregnancy without clinical or obstetric complications were included in the control (normotensive) group (PC). Eleven healthy, non-pregnant women (mean age 30 ± 6 years, NPC) were also evaluated and served as controls.

The inclusion criteria in the study groups encompassed pregnant women with a gestational age of more than 24 weeks and non-pregnant women age-matched to the study group of patients with one or more live births, without a history of complications during or after pregnancy and childbirth.

Pregnant women with chronic hypertension, thyroid diseases, erythrocytopathies, autoimmune diseases, diabetes, or kidney diseases, and patients with twinning or fetal malformation were excluded from the study.

### 4.2. Sample Preparations

Blood samples (12 mL) were collected by intravenous puncture in two tubes (Vacutainer; Becton Dickinson, and Company, Franklin Lakes, NJ, USA) containing K_3_EDTA. Blood samples were centrifuged at 1200 *g* for 15 min, and the yellowish supernatant (plasma and white blood cells) was discarded. The remaining RBCs were resuspended and washed three times in PBS solution (140 mM NaCl, 2.7 mM KCl, 8 mM Na_2_HPO_4_, 1 mM KH_2_PO_4_, and 1 mM EDTA), pH 7.4. Washed RBCs were diluted to 30% hematocrit in PBS buffer and stored at 4 °C for AFM and optical analysis.

### 4.3. Preparation of Spread Cells

A small quantity of washed RBCs (20 µL) was mixed with 20 µL of autologous blood plasma according to the procedure described in [53] and was smeared on poly-L-lysine-coated coverslips by manual spreading.

### 4.4. Optical Microscopy

In the present work, we utilized optical microscopy (suitable for determining the relative abundance of the different morphological types) to characterize the RBC morphology. The quantification of the erythrocytes’ morphological classes during cell aging was carried out with an optical microscope (3D Optical profiler, Zeta-20, Zeta Instruments, Milpitas, CA, USA). All experiments were performed at room temperature. The optical images of the cells were obtained using a magnification 50× objective lens. Extensive mapping was performed for each smear sample. To ensure statistical relevance, at least 500 cells per sample were counted.

### 4.5. AFM Measurements

AFM imaging was done on an atomic force microscope (MFP-3D, Asylum Research, Oxford Instruments, Santa Barbara, CA 93117, USA). All AFM measurements were performed in contact mode at room temperature. Silicon AFM tips (nanosensors, type qp-Bio) of 50 kHz resonance frequency, 0.3 N/m nominal spring constant, and a nominal tip radius of 8 nm (the shape was conical) were used. Morphology observation and morphometric characterization (RBC diameter, height, volume, and membrane roughness) were achieved using Gwyddion and IgorPro 6.37 software. The roughness analysis was performed in two different scanning areas (2.0 × 2.0 µm^2^ and 1.0 × 1.0 µm^2^) of the RBCs. To avoid the effect of cell distortion, we applied a first-order flattening for the selected areas. The R_rms_ value was calculated as the mean square root of the height distribution as follows:

$$Rrms=\sqrt{\sum_{i=1}^{N}\frac{{\left(z_{i}-z_{m}\right)}^{2}}{\left(N-1\right)}}$$

where *N* is the total number of points, *z_i_* is the height of the *i*th point, and *z_m_* is the mean height.

### 4.6. Preparation of Oxidized RBCs

RBCs were isolated from the freshly drawn blood of three healthy women according to the protocol described in Section 4.2. Oxidative stress was induced using hydrogen peroxide (H1009 Sigma-Aldrich Pty Ltd., an affiliate of Merck KGaA, Darmstadt, Germany). RBCs were treated for 4 h at 25 °C with 100 mM or 200 mM H_2_O_2_. Hydrogen peroxide solutions in PBS were prepared from fresh stock solution (1 M) immediately before each experiment to avoid peroxide degradation. The reaction was stopped with 200 µL 10 mM EDTA. Thin smears of untreated and H_2_O_2_-treated RBCs were prepared for AFM analysis, as described in Section 4.3.

### 4.7. Statistical Analysis

Data are presented as mean ± SD (standard deviation). The Mann–Whitney U test was carried out for the comparison of morphometric parameters between the patient groups and the PC and NPC groups (diameter, height, volume, and roughness values). Statistically significant differences between means were assumed significant at *p* < 0.05.

## Figures and Tables

**Table 1:** Characteristics of the studied groups of non-pregnant controls (NPCs), pregnant controls (PCs), and women with preeclampsia (PE). The maternal age, gestational week, mean blood pressure (BP), gestational age (GA) at diagnosis and at delivery, body weight (BW), and main hematological parameters (RBC count; hemoglobin, Hb; hematocrit, Ht; mean corpuscular volume, MCV; mean corpuscular hemoglobin, MCH; mean corpuscular hemoglobin concentration, MCHC; red cell distribution width, RDW; platelet count; aspartate aminotransferase, ASAT; and alanine aminotransferase, ALAT), are presented as mean values and SD.

Characteristic	Reference Values	Reference Values for Pregnant Women	NPC (*n* = 11)	PC (*n* = 12)	PE (*n* = 19)
Maternal age (years)	–		29.8 ± 5.5	27.3 ± 3.4	25.8 ± 2.8
Gestational week (interval)	–		–	24–38	24–36
Mean BP (systolic/diastolic)			111 ± 5/76 ± 4	104 ± 6/72 ± 3	155 ± 11/104 ± 7
GA at diagnosis of PE			-	-	29.3 ± 3.2
GA at delivery			-	39.2 ± 0.4	32.6 ± 2.4
Body weight (kg)				87 ± 7	93 ± 5
RBC count (T/L)	3.6–5.1	4.0–6.2	4.2 ± 0.5	3.8 ± 0.4	3.9 ± 0.5
Hb (g/L)	110–148	120–160	127.0 ± 12	117.3 ± 7.7	112.6 ± 10
Ht (L/L)	0.30–0.46	0.36–0.54	0.376 ± 0.05	0.354 ± 0.02	0.364 ± 0.04
MCV (mmol/L)	82–98	82–100	96 ± 6.0	92.1 ± 6.2	95.2 ± 8.4
MCH (Pg/L)	26.5–32.0	29.0–45.0	39.2 ± 2.0	31.1 ± 2.1	31.4 ± 2.9
MCHC (g/L)	295–360	240–360	331.0 ± 14.0	335.4 ± 5.0	335.4 ± 5.0
RDW (%)	11.5–14.5	11.5–14.5	12.8 ± 1.5	13.8 ± 1.4	13.8 ± 1.6
Platelet count ×10^9^/L	150–400	146–429	301 ± 62	248.6 ± 51.6	242.7 ± 97
ASAT (U/L)	12–38	4–32	18.4 ± 2.8	22.5 ± 3.1	19.7 ± 7.0
ALAT (U/L)	7–41	3–30	17.9 ± 3.3	14.6 ± 1.7	16.3 ± 7.6

**Table 2:** Percentage of RBC morphological classes determined by optical microscopy of freshly isolated cells from non-pregnant controls (NPCs), pregnant controls (PCs), and patients with preeclampsia (PE). Mean ± SD.

Study Group	Morphological Classes (%)		
	Biconcave	Spiculated	Spherocytes
NPC	84 ± 8	16 ± 6	0
PC	79 ± 5	21 ± 4	0
PE	80 ± 9	13 ± 5	7 ± 3

**Table 3:** Morphometric parameters: diameter (D), maximum height (H), volume (V), concavity depth (h_2_), ratio of the maximum height and concavity depth (H/h_2_), and roughness (R_rms_) of freshly isolated RBCs from non-pregnant controls (NPCs) and pregnant controls (PCs), and patients with preeclampsia (PE). R_rms_ values were evaluated from AFM images of a 2 × 2 µm^2^ area.

Group	D (µm)	H (µm)	V (µm^3^)	h_2_ (µm)	H/h_2_	R_rms_ (nm)
NPC	8.1 ± 0.37	0.57 ± 0.05	24.64 ± 3.29	0.50 ± 0.03	1.14 ± 0.11	2.9 ± 0.4
PC	8.2 ± 0.36	0.60 ± 0.07	26.87 ± 3.78	0.51 ± 0.06	1.18 ± 0.09	3.8 ± 0.5
PE	8.5 ± 0.38 *	0.61 ± 0.09	29.82 ± 4.19 *	0.39 ± 0.11 *	2.42 ± 0.82 *	4.7 ± 0.8 *

* Indicates statistical difference at the level of *p* < 0.05, relative to both NPCs and PCs.

## Data Availability

Not applicable.

## Supplementary Materials

The supporting information can be downloaded at: https://www.mdpi.com/article/10.3390/ijms24087100/s1.
