# Supplementary material for: Membrane Lesions and Reduced Life Span of Red Blood Cells in Preeclampsia as Evidenced by Atomic Force Microscopy
Source: Int J Mol Sci. 2023 Apr 12;24(8):7100. doi: 10.3390/ijms24087100 (PMC10138579; doi:10.3390/ijms24087100)
Supplement: Supplementary file 1 [file ijms-24-07100-s001.zip › ijms-2329249-supplementary.pdf]

Membrane lesions and reduced life span of red blood cells in preeclampsia as evidenced by atomic force microscopy

I. Giosheva[1,2], V. Strijkova[1,3], R. Komsa-Penkova[4], S. Krumova[1], A. Langari[1], A. Danailova[1], S.G. Taneva[1], T. Stoyanova[1], L. Topalova[1], Emil Gartchev[2], G. Georgieva[4], S. Todinova[1,]*

[1]Institute of Biophysics and Biomedical Engineering, Bulgarian Academy of Sciences, 1113 Sofia, Bulgaria,
[2]University Obstetrics and Gynecology Hospital "Maichin Dom", 1431 Sofia, Bulgaria,
[3]Institute of Optical Materials and Technologies "Acad. Yordan Malinovski", Bulgarian Academy of Sciences, 1113 Sofia, Bulgaria,
[4]Department of Biochemistry, Medical University, 5800, Pleven, Bulgaria

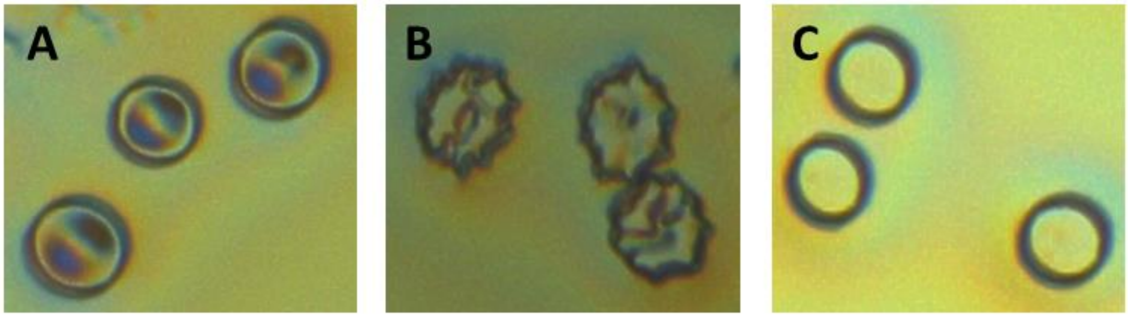

Figure S1. Typical 2D images of the main RBC morphologies recorded by optical microscopy: (A) biconcave; (B) spiculated and (C) spherical. The images were captured using a magnification 50× objective lens.

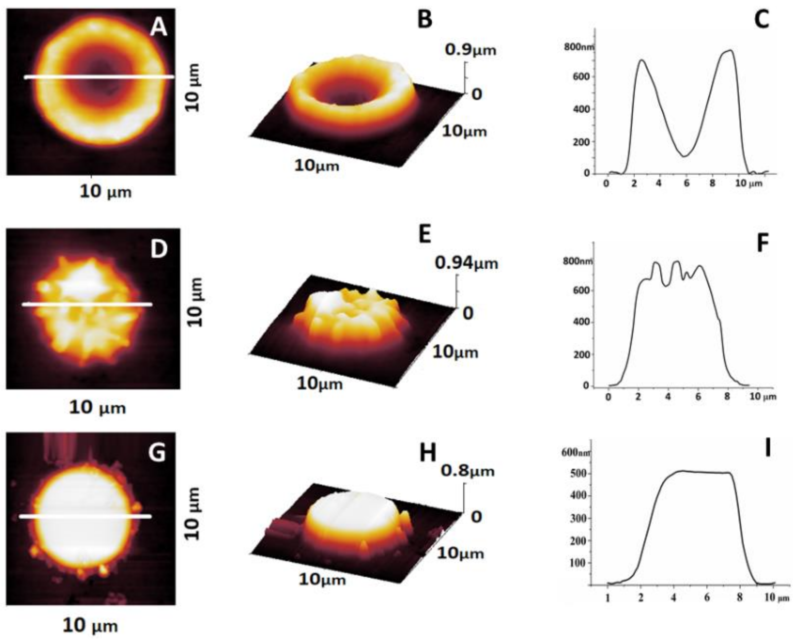

Figure S2. Typical 2D (A,D,G) and 3D (B,E,H) AFM images of the main RBC morphologies: (A, B) biconcave; (D, E) spiculated; and (G, H) spherical. The corresponding cross-sectional profiles of the cells defined by the white line of the 2D AFM images are given on panels C, F, and I.

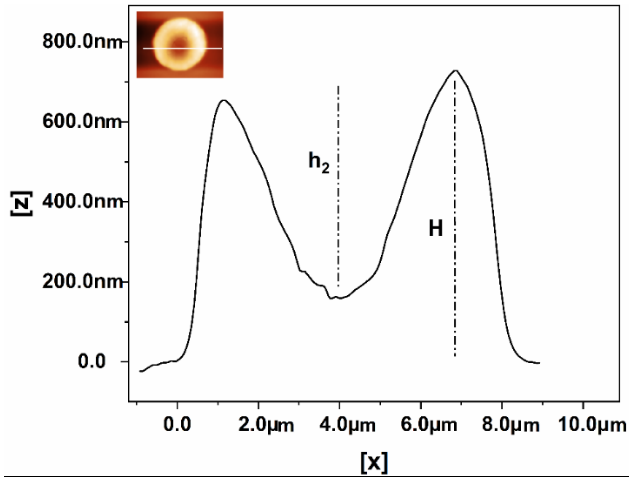

Figure S3. Example of a cross-sectional profile of biconcave red blood cell. The profile corresponds to the white line crossing the cell in the inset. The dashed lines indicate the maximum height (H) and the depth of concavity (h_2) of the cell.

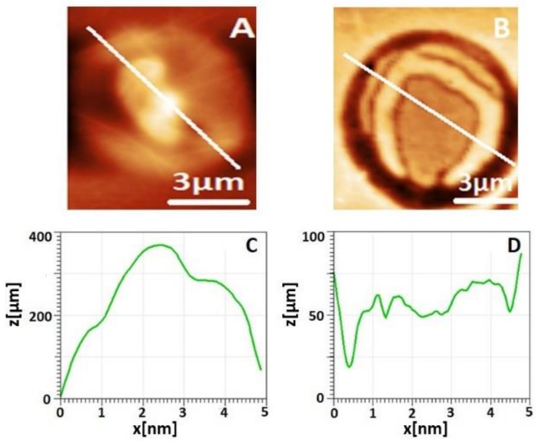

Figure S4. Selected 2D images of RBCs isolated from patients with PE exhibiting abnormal morphology (A) and “ghosts” (B). The corresponding cross-sectional profiles of the cells defined by the white line of the AFM images are given on panels C and D.
